# The Trouble with Trials: Systematic Review and Meta-Analysis of Randomized Controlled Trials Comparing Stereotactic Radiosurgery, Whole Brain Radiotherapy, and Observation for Resected Metastatic Brain Disease

**DOI:** 10.3390/cancers18071149

**Published:** 2026-04-02

**Authors:** Khalil St Brice, Baylee Stevens, Avital Perry, Victor M. Lu, Shearwood McClelland, Tyler Gunter, Christopher S. Graffeo

**Affiliations:** 1 Department of Neurosurgery, University of Oklahoma, Oklahoma City, OK 73104, USA; stbricekhalil@gmail.com (K.S.B.);; 2 Department of Neurosurgery, Sheba Medical Center, Tel Aviv 52621, Israel; 3 Department of Neurosurgery, University of Miami, Miami, FL 33136, USA; 4 Department of Radiation Oncology, University of Oklahoma, Oklahoma City, OK 73104, USA

**Keywords:** stereotactic radiosurgery, whole brain radiotherapy, brain metastases, systematic review, meta-analysis, overall survival, cognitive decline

## Abstract

**Background**: Brain metastases are a major driver of cancer-associated morbidity and mortality, occurring in 20–40% of all malignancies. **Objective**: Systematic review and meta-analysis of randomized controlled trials (RCTs) comparing whole brain radiotherapy (WBRT), stereotactic radiosurgery (SRS), or observation after resection of 1–4 brain metastases. **Methods**: PRISMA-compliant search of Embase and MEDLINE from inception to August 2024. Overall survival (OS), cognitive decline (CD), surgical bed control (SBC), and intracranial control (IC) were assessed by pooling hazard ratios (HR) or 12-month post-SRS odds ratios (OR) via meta-analysis of proportions with random-effects modeling. **Results**: Of 1319 unique, English-language abstracts, 37 underwent full-text review, and 7 were included, representing 812 patients in three paired comparisons: WBRT vs. observation (4 RCTs, *n* = 545), WBRT vs. SRS (2 RCTs, *n* = 253), and SRS vs. observation (1 RCT, *n* = 132). Pooled HR were not significant for OS (WBRT vs. observation, HR = 1.01, 95% CI = 0.87–1.18; WBRT vs. SRS, 0.77, 95% CI = 0.47–1.26) or CD (WBRT vs. observation, HR = 0.98, 95% CI = 0.81–1.17; WBRT vs. SRS, 1.31, 95% CI = 0.48–3.60). Pooled OR favored WBRT over both observation and SRS with respect to SBC (SBC: WBRT vs. observation, OR = 1.35, 95% CI = 1.05–1.74; WBRT vs. SRS, OR = 1.46,95% CI = 1.00–2.13) and IC (IC: WBRT vs. observation, OR = 1.22, 95% CI = 1.06–1.40; WBRT vs. SRS, OR = 1.22, 95% CI = 1.03–1.59). GRADE assessment demonstrated very low to moderate certainty for all reported outcomes. **Conclusions**: Even among RCTs, the best available evidence regarding adjuvant treatment for oligometastatic brain disease is heterogeneous, imprecise, and inconclusive. WBRT improves SBC and IC, but not OS. SRS may decrease CD risk, which requires validation in double-blind RCTs incorporating precise neurocognitive metrics.

## Simple Summary

Brain metastases occur in 20–40% of all malignancies and represent a leading driver of cancer-associated morbidity and mortality. Surgical resection is frequently offered for large or symptomatic lesions, but the optimal adjuvant strategy following resection remains actively debated. We conducted the first meta-analysis of randomized controlled trials comparing the three major postoperative strategies for resected brain metastases, whole brain radiotherapy, stereotactic radiosurgery, and observation, with specific attention to primary outcomes of overall survival (OS) and cognitive decline (CD), and secondary outcomes of surgical bed control (SBC) and intracranial control (IC). We found that while both radiotherapy modalities improve local and intracranial disease control compared to observation, neither confers a demonstrable survival advantage. Stereotactic radiosurgery may decrease the risk of cognitive decline. Although definitive evidence remains lacking, based on our interpretation of the strongest available data, adjuvant SRS represents a clinically reasonable and preferred postoperative option for patients following surgical resection of oligometastatic brain disease. These findings expose fundamental limitations in the existing trial literature and reinforce the need for more rigorously designed randomized studies incorporating contemporary neurocognitive endpoints and neuroprotective treatment standards.

## 1. Introduction

Brain metastases are a major driver of cancer-associated morbidity and mortality, affecting 20–40% of patients with malignancies [1,2]. Treatment strategies for brain metastases include surgical resection, irradiation (via whole brain radiotherapy [WBRT] or stereotactic radiosurgery [SRS]), systemic therapies, and expectant management, often in various combinations. Although small lesions identified on surveillance imaging may be managed via front-line irradiation or systemic therapy, surgical resection is frequently offered for large or symptomatic lesions. Surgical resection may also provide a survival benefit, particularly for solitary metastases causing mass effect, in oligometastatic disease (defined as ≤4 intracranial metastases), or in cases where the dominant lesions are in critical locations or associated with marked cerebral edema [3,4,5,6,7,8].

Adjuvant WBRT following surgical resection decreases the risk of marginal recurrence at the surgical site and improves overall intracranial disease control. However, multiple randomized trials, observational studies, and meta-analyses have failed to show an improvement in overall survival (OS) after WBRT when compared to surgery alone (e.g., observation). Furthermore, WBRT has been consistently associated with an increased risk of cognitive decline (CD) and numerous other toxicities, including alopecia, fatigue, and xerostomia [2,9,10,11,12,13,14,15,16,17,18]. While WBRT significantly enhances intracranial control (IC) and reduces local recurrence, these improvements in disease control have not been associated with a definitive improvement in survival benefit or reduction in cognitive decline.

In an attempt to minimize the toxic effects of post-operative WBRT, SRS to the resection bed has become increasingly adopted as an alternative adjuvant treatment for oligometastatic brain disease [9,12,16,17,19]. Traditional postoperative SRS involves a single-fraction, frame-based technique that delivers highly conformal therapeutic radiation; it is considered a cognition-sparing modality based on several decades of observational and randomized data [9,12,16,20]. More modern postoperative SRS (particularly for resection cavities exceeding 3 cm in maximum diameter) has involved multiple (typically 3–5) fractions of frameless (mask-based) treatment to reduce the risk of toxicity (particularly radiation necrosis) and increase local control for larger resection cavities [21,22]. In recent years, at least three randomized trials have been published that incorporate formal neurocognitive or functional assessments as study endpoints, highlighting the need for an updated consolidation of the best available evidence on this key clinical question [9,11,16,23].

Correspondingly, the aim of this study was to conduct a systematic review and meta-analysis of randomized controlled trials comparing the three major postoperative management strategies for metastatic brain disease—WBRT, SRS, or observation—with specific attention to the critical outcomes, overall survival (OS) and cognitive decline (CD).

## 2. Methods

### 2.1. Search Strategy

Under the supervision of a research librarian, we designed our search strategy using the PICOS question format: *Do patients with surgically resected metastatic brain disease (Population) treated with SRS (Intervention), WBRT (Comparator 1), or observation (Comparator 2) differ in OS and/or CD (Outcome), based on RCT evidence (Study Type).* The review was conducted in compliance with the Preferred Reporting Items for Systematic reviews and Meta-Analyses (PRISMA) guidelines [24] (Supplement S5). Electronic searches were conducted via Ovid MEDLINE (Supplement S1) and Embase (Supplement S2), using combinations of MeSH terms and keywords, covering the time period from database inception through August 2024. This was not prospectively registered in PROSPERO.

### 2.2. Selection Criteria

Abstracts for all retrieved articles were independently screened against prespecified criteria by two investigators (CSG, AP) for identification of candidate articles. Studies were included if they met the following criteria: (1.) randomized controlled trial; (2.) direct comparison of WBRT or SRS with each other or with observation; (3.) enrollment of patients with oligometastatic brain disease; and (4.) reporting a hazard ratio (HR) effect size for OS as a study endpoint. Articles without English language translations were excluded, although no candidate abstracts flagged for full-text review required full-text translation. A single definition of brain metastasis was not adhered to, so long as the included studies specified a definition for patients included in their protocol. Trials evaluating multiple interventions were eligible if data could be distinctly attributed to individual trial arms and at least two arms satisfied inclusion criteria (e.g., trials in which only two of three groups underwent front-line resection were included if arm-level data were clearly reported). All abstracts identified as candidates for inclusion by either reviewer underwent full-text review by both reviewers.

### 2.3. Data Extraction

Two investigators independently reviewed full-length articles and extracted relevant study outcomes from tables, figures, or texts. Discrepancies were resolved by consensus. Whenever possible, effect sizes were directly abstracted; when required, HRs or other estimates were derived based on available data and satisfaction of appropriate statistical assumptions [25,26,27].

Associations between treatment arms and OS were expressed as the hazard of death after Treatment A relative to Treatment B (e.g., WBRT vs. observation; SRS vs. WBRT; SRS vs. observation). CD was similarly reported as the pooled hazard ratio for cognitive decline comparing treatment groups. Preferred definitions of CD were those based on validated, comprehensive cognitive batteries administered by trained and certified neuropsychiatric staff. Measures based on validated but less comprehensive training (e.g., Mini-Mental Status Exam (MMSE), Medical Research Council [MRC] neurologic status) were accepted but incurred a moderate downgrading in GRADE assessments. Study-specific or functional-status-based definitions of CD, (e.g., Karnofsky Performance Status, WHO scale, modified Rankin Scale), when previously validated as indirect indicators of post-WBRT cognitive decline, were included but received the maximum downgrading in evidence quality assessments [28].

Surgical bed control (SBC) and intracranial control (IC) were defined as the development of new enhancing lesions post-SRS within or immediately adjacent to the resection cavity (SBC) or beyond the treatment field (IC). SBC and IC incidences at 12 months were abstracted and pooled for meta-analysis, with results reported as the odds ratio (ORs) of surgical bed or intracranial failure. Trials incorporating blinded neuroradiology review were preferred; protocols lacking explicit blinding but providing clear, compliant definitions of SBC/IC were included but down-graded on evidence quality assessment for risk of bias.

### 2.4. Meta-Analysis

Studies were grouped according to the treatment comparisons within each RCT (WBRT vs. observation; SRS vs. WBRT; SRS vs. observation). For primary outcomes (OS and CD), HRs with 95% confidence intervals were logarithmically transformed and pooled using a meta-analysis of proportions technique to calculate summary statistics. For secondary outcomes (SBC and IC), 12-month ORs with 95% CI were calculated and pooled. Random-effects models were used for analyses to account for expected clinical and methodological heterogeneity. Heterogeneity was assessed using I^2^. All statistical tests were two-sided, with statistical significance defined as α of 0.05. Analyses were conducted using STATA 14.1 (StataCorp, College Station, TX, USA) [29].

### 2.5. Evidence Quality & Bias Assessments Methods

The certainty of evidence for primary outcome was evaluated using the Grading of Recommendations, Assessment, Development and Evaluations (GRADE) framework, with results summarized by outcome and comparison group. Risk of bias was assessed according to the Cochrane Collaboration guidelines and reported for each outcome both by study and in pooled summary graphs [30]. GRADE and bias assessments were completed independently by two investigators (CSG, AP), with disagreements resolved by consensus.

Consideration was given to assessment of publication bias using funnel plots for OS and CD by comparison; however, given the small number of studies ultimately identified as meeting criteria (*n* = 7, with ≤3 per comparison group), and the decision to use a random-effect model for meta-analysis, these were considered not meaningfully interpretable, and correspondingly were not reported. Similar consideration was given to the inclusion of Egger’s linear regression and Begg’s correlation tests for assessment of small study bias, both of which were deferred on the basis of inadequate interpretability [31,32].

## 3. Results

### 3.1. Systematic Literature Review

**Figure 1:**
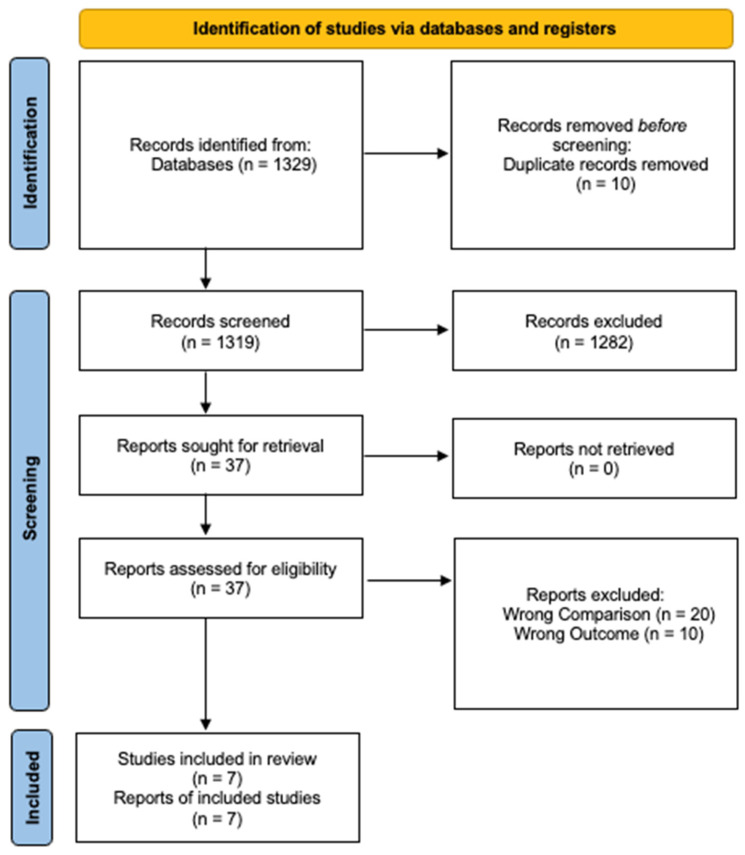
PRISMA diagram.

After de-duplication, initial literature search identified 1,319 unique English-language publications for abstract review (Figure 1). Of these, 37 were selected for full-text review based on predefined criteria, resulting in the final inclusion of 7 publications [9,11,12,14,16,18].

### 3.2. Overview of Included Studies

The seven included RCTs comprised a total of 812 patients. Four studies compared WBRT vs. observation (*n* = 545), two compared SRS vs. WBRT (*n* = 253), and one compared SRS vs. observation (*n* = 132) (Table 1 and Table 2). Six of the seven studies reported an acceptable metric for CD, and all studies reported outcomes related to surgical bed control (SBC) and intracranial control (IC). No trial reported a statistically significant difference in OS between treatment arms. Brown et al. reported a significantly decreased hazard of CD after SRS compared to WBRT (HR = 0.47, 95% CI = 0.35–0.63) [9].

### 3.3. WBRT Versus Observation

**Figure 2:**
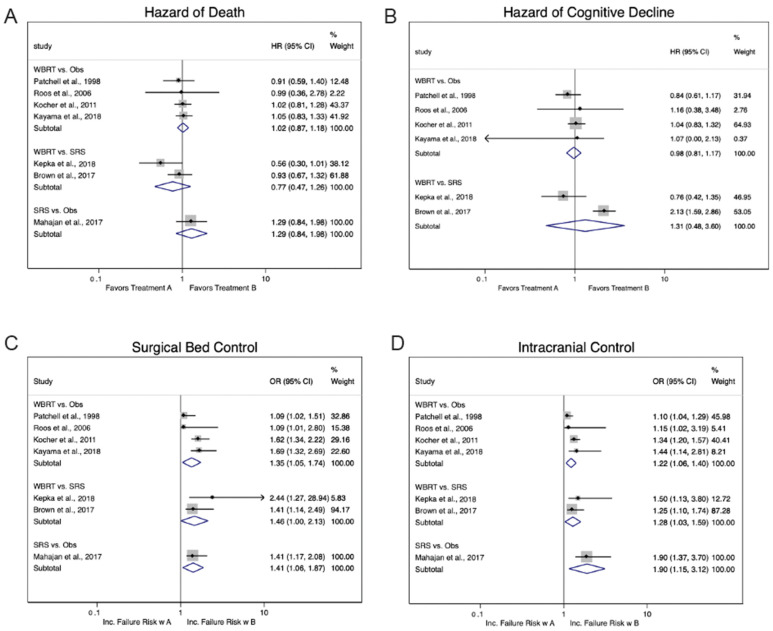
Forest plots for (**A**) overall survival [6,9,11,13,14,16,18], (**B**) cognitive decline [6,9,11,13,14,18], (**C**) surgical bed control [6,9,11,13,14,16,18], and (**D**) intracranial control [6,9,11,13,14,16,18]. Plots demonstrate the multiple comparisons for each outcome in parallel; however, given the differences in treatment arms by comparison, an overall pooled summary was not performed. WBRT = Whole brain radiotherapy, Obs = Observation, SRS = Stereotactic radiosurgery, HR = Hazard ratio, CI = Confidence Interval.

Four RCTs compared adjuvant WBRT vs. observation [14,18]. All four studies reported sufficient data for assessment of both OS and CD, however, three relied on imprecise CD metrics. In pooled analysis for OS, the HR for death after WBRT compared with observation was 1.02 (95% CI = 0.87–1.18; Figure 2A), with no detectable heterogeneity (I^2^ = 0.0%, P_het_ = 0.96). The pooled HR for CD was similarly nonsignificant at 0.98 (95% CI = 0.81–1.17; Figure 2B; I^2^ = 0.0%, P_het_ = 0.75). Both SBC and IC favored WBRT, with pooled ORs for local and intracranial failure after observation of 1.35 (95% CI = 1.05–1.74; Figure 2C) and 1.22 (95% CI = 1.06–1.40; Figure 2D), respectively. Heterogeneity was moderate for SBC (I^2^ = 64.5%, P_het_ = 0.04) and lower for IC (I^2^ = 44.5%, P_het_ = 0.15).

### 3.4. WBRT Versus SRS

Two RCTs compared postoperative SRS with WBRT [9,13]. Both used high-quality neurocognitive batteries to assess CD, and one trial incorporated blinding of the assessing neuropsychiatrist to patient allocation. The pooled HR for death after WBRT vs. SRS was 0.77 (95% CI = 0.47–1.26; Figure 2A), with moderate heterogeneity (I^2^ = 54.0%, P_het_ = 0.14). For CD, the pooled analysis for decline after WBRT vs. SRS was 1.31 (95% CI = 0.48–3.60; Figure 2B), with significant heterogeneity (I^2^ = 89.7%, P_het_ < 0.01). As with WBRT vs. observation, SBC and IC both favored WBRT. The pooled ORs for local and intracranial failures after SRS were 1.46 (95% CI = 1.00–2.13; Figure 2C) and 1.22 (95% CI = 1.03–1.59; Figure 2D), respectively. No heterogeneity was detected in these analyses (I^2^ = 0.0%, P_het_ = 0.51 and P_het_ = 0.59).

### 3.5. SRS Versus Observation

To date, only one published RCT has compared postoperative SRS with observation, precluding the possibility of meta-analysis [16]. In this trial, the HR for death after SRS vs. observation was 1.29 (95% CI = 0.84–1.98; Figure 2A), a nonsignificant difference. No formal CD assessments were reported. SBC and IC both favored SRS, with ORs for local and intracranial failures after observation of 1.41 (95% CI = 1.17–2.08; Figure 2C) and 1.90 (95% CI = 1.37–3.70; Figure 2D), respectively.

### 3.6. Evidence Quality & Bias Assessments Results

GRADE assessments were conducted for each comparison eligible for meta-analysis, with certainty of evidence across outcomes ranging from very low to moderate (Table 3). The absence of provider blinding in all included trials resulted in universal downgrading for risk of bias. Both CD comparison groups were further downgraded for inconsistency, reflecting the variability in neurocognitive testing. The WBRT vs. observation comparison was further downgraded for imprecision, as all four studies relied on lowest-quality CD metrics. Comparisons between WBRT and SRS were also downgraded for imprecision due to the small number of studies (*n* = 2).

Across all seven studies, absent, poor, or incomplete allocation concealment protocols contributed to moderate-to-high overall risk-of-bias (Supplements S3 and S4). With respect to OS, the objective nature of the outcome minimized detection bias, resulting in moderate risk of bias ratings for four studies and high risk for three (Supplement S3). Only one study incorporated a preferred cognitive battery and a blinded outcome assessment, resulting in a moderate risk of bias rating for CD; the remaining five trials reporting CD outcomes were rated as having high risk of bias (Supplement S4).

## 4. Discussion

Although best management of brain metastases after surgical resection remains a complex and actively evolving area of investigation, the major unanswered questions can be reduced to two core considerations: *Does adjuvant therapy confer benefit, and if so, which modality provides the greatest effect?* These questions must be addressed with respect to both survival and cognition—outcomes that are often at odds when treatment involves WBRT.

In this meta-analysis, limited to RCTs directly comparing WBRT, SRS, or observation, we failed to demonstrate a significant difference in either primary endpoint, OS and CD. The most straightforward interpretation is that no such differences exist, and that these treatments are equivalent with respect to survival and cognition. While there is a gratifying simplicity to this view, it also requires considerable skepticism. Across all trials, we observed large and consistent differences in SBC and IC, indicating meaningful variation in the biological impact of these interventions on target tissue. It is therefore difficult to reconcile the absence of any corresponding effect on normal brain tissue, particularly regarding cognition. Moreover, the significantly decreased risk of CD after SRS compared with WBRT reported by Brown et al., supported by strong trial methodology and neurocognitive assessment, makes dismissal of a true cognitive advantage even more challenging [9].

An alternative and more plausible explanation draws on the elements of substantial confounding, bias, and methodological limitations present across the seven included RCTs, compounded by the small number of studies included. Several observations support this perspective as a more likely interpretation.

### 4.1. Challenges in OS Assessment

With respect to OS, most included RCTs were not powered to detect modest but clinically meaningful differences between treatment arms. Although OS is arguably the outcome of greatest importance to patients, it is also among the most challenging to evaluate in comparative trials of local therapy. Trials powered for OS require large sample sizes and substantial follow-up, particularly when the expected between-group survival difference is small.

Practical and structural pressures further complicate OS assessment. Industry sponsors, institutions, and patient advocacy groups often prioritize expedited study completion and timely dissemination of results that may open avenues to new treatments. These incentives favor the use of surrogate endpoints, such as SBC or IC, that are more cost effective to assess, require smaller cohorts, and can deliver actionable results on a relatively short timeline.

Confounding introduces an additional challenge. Patients with resected oligometastatic brain disease originate from a wide host of primary pathologies, each with its own natural history, prognosis, pattern of systemic spread, and options for salvage therapy. This heterogeneity decreases the ability of any trial to detect treatment effects applicable across all disease types. Thus, while the failure to demonstrate an all-comers benefit for SRS in this umbrella population may seem disappointing, it is not unexpected and does not preclude the possibility that specific histology- or phenotype-defined subgroups may derive meaningful benefit when evaluated in isolation.

### 4.2. Challenges in CD Assessment

While OS as an endpoint is unambiguous, its assessment requires longer follow-up, an adequate patient cohort, and a robust approach to account for confounding factors. In contrast, CD tends to occur earlier in the post-treatment period and, based on observational data, shows larger expected differences between treatment groups. This makes CD easier to detect with statistical significance in smaller sample sizes. However, CD is an inherently inconsistent outcome, further complicated by the use of heterogeneous and imprecise metrics for assessing cognitive function. In our sample of six RCTs reporting CD, three used unfavorable or imprecise metrics, while the other three employed validated neurocognitive instruments, though two of these lacked assessor blinding to treatment allocation. It should be noted that these two categories, formal neuropsychological testing, and validated but less comprehensive measures (e.g., MMSE, MRC neurologic status), are not equivalent constructs; the former captures objective cognitive domains while the latter reflects broader functional status and may be influenced by physical or systemic factors unrelated to cognition. This distinction was pre-specified in our data extraction protocol, where less comprehensive measures incurred a moderate downgrading in GRADE assessments for indirectness, and is reflected in the very low certainty ratings assigned to both CD comparison groups.

Further, although Brown et al. reported a robust methodology with assessor blinding, the clinical team responsible for patient treatment and follow-up was unblinded [9]. This lack of blinding, although a potential source bias, must be considered in the context of the logistical challenges involved in delivering WBRT and SRS. The absence of any physician or patient masking in these trials demonstrates a fundamental challenge inherent to the differing logistics of these two treatment modalities. Future trials should strongly consider using sham comparisons in lieu of true observation groups to mitigate such biases.

Of particular note, the included WBRT trials did not co-administer memantine, a neuroprotective agent that has shown promising preliminary results as a cognition sparing modality [33]. Contemporary randomized data demonstrate that memantine, an NMDA-receptor antagonist, not only mitigates cognitive decline during WBRT but also improves patient-reported cognitive and neurological symptoms. In the phase III CC001 trial, memantine was mandated in both arms (WBRT + memantine vs. HA-WBRT + memantine) and demonstrated significant cognitive benefits without negatively affecting OS or intracranial progression-free survival [33]. Mechanistically, memantine attenuates increases in vascular permeability in normal-appearing white matter and is associated with better verbal fluency, supporting its potential radioprotective effect [34].

The hippocampal avoidance (HA) technique, which limits radiation dose to the hippocampal dentate gyri while treating the remaining brain, has also demonstrated cognitive benefits. In CC001, combining HA with WBRT and memantine further reduced the risk of cognitive failure, with no detriment to OS or intracranial progression-free survival. These cognitive and patient-reported benefits were sustained beyond one year [33,34]. Even without memantine, HA-WBRT has shown better memory preservation compared to conformal WBRT, again without compromising intracranial control or survival [35]. Evolving approaches, such as HA-WBRT with a simultaneous integrated boost, also appear feasible [36]. Taken together, these findings suggest that historical trials of WBRT conducted without memantine or hippocampal avoidance may have overstated the cognitive penalties associated with contemporary WBRT. This has direct implications for the interpretation of our pooled CD results, as none of the included WBRT trials incorporated HA-WBRT or mandated memantine use, meaning our analysis reflects a treatment that is increasingly considered substandard in routine clinical practice. As such, the cognitive penalties associated with WBRT observed in these trials may not be fully representative of outcomes achievable with contemporary neuroprotective approaches, further underscoring the need for updated randomized comparisons using modern WBRT standards.

Finally, the failure to demonstrate a significant difference in CD between WBRT and SRS should be interpreted with considerable skepticism, given that only two studies contributed to this subgroup analysis. One of these used the MMSE to assess CD, rather than formal neuropsychiatric testing, and reported an imprecise effect size with a wide, non-significant confidence interval [12]. Both studies exhibited statistically significant heterogeneity, and a GRADE assessment indicated low or very low certainty, further emphasizing the unreliable nature of this subgroup analysis. Correspondingly, we interpret the pooled results with extreme caution and place more emphasis on the results from the single, more robust, less confounded RCT [15]. The high between-study heterogeneity observed for CD in the WBRT vs. SRS comparison (I^2^ = 89.7%) can be attributable to the fundamental incompatibility of the two included studies’ cognitive assessment instruments: Brown et al. employed a comprehensive, multi-domain, assessor-blinded neuropsychological battery, while Kepka et al. relied on the MMSE, a brief screening tool with well-documented ceiling effects, embedded within a composite neurological endpoint. When contributing studies differ not only in their patient populations and treatment protocols but in the construct being measured, the resulting heterogeneity most likely reflects methodological incompatibility, and the pooled estimate should be interpreted with corresponding caution.

### 4.3. The Question of Control

Disease recurrence after resection of brain metastases most commonly occurs at the surgical bed. Although both WBRT and SRS have shown significant improvements in local control compared to resection alone, durable local control has not been associated with improved OS in RCTs assessing intracranial irradiation by any modality [4,9,11,13,37,38]. This repeatedly observed finding raises an important question: Why treat the resection bed in the perioperative period, rather than waiting to intervene at the time of recurrence? To address this, two clinical concepts warrant consideration, particularly in the absence of an OS benefit.

First, the indication for resection is highly pertinent within the context of resected metastatic disease. Although many instances of oligometastatic disease may be reasonably managed with SRS alone, those cases that are selected for surgery are typically larger, located in more critical regions, or causing symptoms that are adversely impacting the patient’s quality of life [4]. Correspondingly, the risks associated with disease recurrence are increased, particularly since lesion size is the strongest predictor of recurrence after resection of brain metastases [13,15,39,40]. At present, data on the quality-of-life benefits of upfront SRS to the resection bed are lacking; however, given SRSs favorable toxicity profile, early treatment to potentially minimize the risk of clinically debilitating symptoms is a reasonable consideration. The same rationale may be extended to WBRT, although at least one RCT has demonstrated a significantly increased risk of CD after WBRT compared to SRS [9].

Second, the paradigm shift in the management of brain metastases, from a leading cause of mortality to a chronic but manageable aspect of the disease, has dramatic implications for the future of cancer care [1,41,42]. Although current systemic treatments for metastatic disease provide only modest improvements in OS, a number of promising therapies are actively being investigated, including CAR T-cell therapies, immunotherapies, targeted therapies, checkpoint inhibitors, and other novel chemotherapeutics [43,44,45]. In this context, although postoperative adjuvant SRS may not provide an OS benefit unto itself, it could help maintain intracranial disease control, potentially allowing a larger number of patients to benefit from improved systemic therapies in the future, as well as participate in clinical trials aimed at answering these critical questions [9]. The shifting paradigm in postoperative SRS from single-fraction frame-based treatment to multi-fraction masked-based treatment may have been underestimated by our analysis, which focused predominantly on single-fraction postoperative SRS, which yields inferior local control for large resection cavities compared to multi-fraction SRS. The predominance of single-fraction approaches in the trials included in this analysis may therefore have artificially compressed the apparent benefit of SRS relative to both WBRT and observation, given the elevated risk of radiation necrosis and reduced local control efficacy associated with single-fraction treatment at larger cavity volumes [46]. It is plausible that the routine adoption of fractionated SRS (3–5 fractions), where fractionated regimens have demonstrated better local control and lower rates of radiation necrosis, would yield a more favorable toxicity and efficacy profile than what is reflected in these trials, and future RCTs should incorporate fractionated SRS as the standard intervention arm [46]. Underlying all of these considerations is a biological reality that deserves explicit acknowledgment: the consistent failure of adjuvant irradiation to extend OS in this population is, in large part, a reflection of where these patients actually die. In patients with resected brain metastases, the majority of deaths are driven by systemic disease progression rather than intracranial recurrence, and improved locoregional control of the surgical bed, however meaningful for neurological function and quality of life, is unlikely to confer a survival advantage in a population whose prognosis is ultimately dictated by the trajectory of their extracranial disease.

### 4.4. Strengths & Limitations

This meta-analysis is strengthened by its focus on RCTs, a methodological choice that enhances the reliability of our conclusions, particularly when compared to preceding observational studies or meta-analyses derived from observational data. The study also adhered to the PRISMA and GRADE guidelines, which add additional credibility to its findings. Finally, despite the widespread use of WBRT in the trials reviewed, we chose not to pool the non-WBRT arms, prioritizing a more clinically relevant and discriminatory analysis over a larger study sample size.

However, the study is subject to several major limitations, ultimately reflecting the limited number of trials on post-resection adjuvant irradiation treatments. Only seven studies met inclusion criteria, all of which were small, heterogeneous, and marked by moderate-to-severe risks of bias. Furthermore, due to heterogeneity in the treatment arms compared, the groups eligible for meta-analysis consisted of only four and two studies, respectively, well below the preferred range of five to ten studies per subgroup. Due to the small number of studies and the potential variability in treatment effects between inconsistently designed trials, the certainty of the pooled outcomes is very low, as reinforced by both our GRADE assessment and the I^2^ values >25% in multiple comparisons.

## 5. Conclusions

We report the first meta-analysis of RCTs assessing WBRT, SRS, and observation in the postoperative adjuvant setting, with respect to primary outcomes (OS and CD) and secondary outcomes (SBC and IC). Our study found no significant difference in the relative hazard of death or cognitive decline between WBRT, SRS, or observation, based on the notably limited available literature. Despite this, our analysis successfully highlights several key shortcomings shared by nearly all of the preceding clinical trials in this space, underscoring the need for a more patient-centric approach in future trial design that prioritizes clinically relevant primary outcomes and appropriate study powering. Although definitive evidence remains lacking, based on our interpretation of the strongest available data, adjuvant SRS represents a clinically reasonable and preferred option for most patients following surgical resection of oligometastatic brain disease, given the potential short- and long-term benefits previously observed including its favorable toxicity profile, and the reduced patient burden associated with fewer treatment visits compared to WBRT, factors that retain meaningful value even in the absence of high-certainty randomized evidence. Future trial efforts in this space should provide adequate power for definitive OS assessment; incorporate blinded assessments using advanced neuropsychiatric tools for CD; and incorporate sham-controlled observation. Additionally, randomized trials of single versus multi-fraction postoperative SRS will provide additional details, as this has become an increasing paradigm shift in radiation oncology management of this patient population. Perhaps most important, focused randomized assessment of SRS versus WBRT with co-administration of memantine (rather than WBRT alone) and hippocampal-sparing protocols is mandatory, in order to better interrogate whether the distant disease control benefits conferred by WBRT can be achieved in a manner that achieves or at least approaches the neurocognitive benefits of SRS.

## Figures and Tables

**Table 1:** Overview of included studies & primary outcomes.

Study	Design	Total Enrollment (A vs. B)	Median Age (Range)	Female Sex (%)	HR for Death (95% CI) *	HR for Cognitive Decline (95% CI) ^
Patchell et al., 1998 [6]	WBRT vs. Obs	95 (49 vs. 46)	59 (38–80) ^†^	40 (42%)	0.91 (0.59–1.40)	0.84 (0.61–1.17)
Roos et al., 2006 [18]	WBRT vs. Obs	19 (10 vs. 9)	58 (27–74) ^†^	5 (26%)	0.99 (0.36–2.78)	1.16 (0.38–3.48)
Kocher et al., 2011 [14]	WBRT vs. Obs	160 (81 vs. 79)	60 (26–81)	124 (35%)	1.02 (0.81–1.28)	1.04 (0.83–1.32)
Kayama et al., 2018 [11]	WBRT vs. Obs	271 (137 vs. 134)	61 (28–79) ^†^	136 (50%)	1.05 (0.81–1.33)	1.07 (0.005–2.13)
Kepka et al., 2018 [13]	WBRT vs. SRS	59 (30 vs. 29)	60 (30–78)	33 (56%)	0.56 (0.30–1.01)	0.76 (0.42–1.35)
Brown et al., 2017 [9]	WBRT vs. SRS	194 (96 vs. 98)	61 (54–68) ^††^	98 (51%)	0.93 (0.67–1.32	2.13 (1.59–2.86)
Mahajan et al., 2017 [16]	SRS vs. Obs	132 (63 vs. 65)	57 (20–80) ^†^	60 (47%)	1.29 (0.84–1.98)	NR

WBRT = Whole brain radiotherapy, Obs = Observation, SRS = Stereotactic radiosurgery, HR = Hazard ratio, CI = Confidence Interval, NR = Not reported, CD = Cognitive decline; ^†^—Median values approximated from incomplete data; ranges accurate; ^††^—Approximate pooled median and IQR; *—HR for OS represents hazard of death, treatment A vs. B; ^—HR for CD represents hazard of decline, treatment A vs. B.

**Table 2:** Surgical bed control & intracranial control.

Study	Design	OR SBC (95% CI) *	OR IC (95% CI) ^
Patchell et al., 1998 [6]	WBRT vs. Obs	1.09 (1.02–1.51)	1.10 (1.04–1.29)
Roos et al., 2006 [18]	WBRT vs. Obs	1.09 (1.01–2.80)	1.15 (1.02–3.19)
Kocher et al., 2011 [14]	WBRT vs. Obs	1.62 (1.34–2.22)	1.34 (1.20–1.57)
Kayama et al., 2018 [11]	WBRT vs. Obs	1.69 (1.32–2.69)	1.44 (1.14–2.81)
Kepka et al., 2018 [13]	WBRT vs. SRS	2.44 (1.27–28.94)	1.50 (1.13–3.80)
Brown et al., 2017 [9]	WBRT vs. SRS	1.41 (1.14–2.49)	1.25 (1.10–1.74)
Mahajan et al., 2017 [16]	SRS vs. Obs	1.41 (1.17–2.08)	1.90 (1.37–3.70)

OR = Odds ratio, SBC = Surgical bed control, IC = intracranial control, WBRT = Whole brain radiotherapy, CI = Confidence Interval, *—ORs reported as odds of surgical bed failure at 12 months, treatment B vs. A ^—ORs reported as odds of intracranial failure at 12 months, treatment B vs. A.

**Table 3:** GRADE assessment for reported outcomes.

Outcome (Groups)	*n*	Summary Statistic	Pooled Statistic (95% CI)	Type of Evidence	Risk of Bias	Inconsistency	Indirectness	Imprecision	Publication Bias	Overall GRADE
OS (WBRT vs. Obs)	4	HR	1.02 (0.81–1.18)	RCT	−1	0	0	0	0	⨁⨁⨁ Moderate
OS (SRS vs. WBRT)	2	HR	0.77 (0.47–1.26)	RCT	−1	0	0	−1	0	⨁⨁ Low
CD (WBRT vs. Obs)	4	HR	0.98 (0.81–1.17)	RCT	−1	−1	0	−1	0	⨁ Very Low
CD (SRS vs. WBRT)	2	HR	1.31 (0.48–3.60)	RCT	−1	−1	0	−1	0	⨁ Very Low

⨁⨁⨁ Moderate certainty of evidence; ⨁⨁ Low certainty of evidence; ⨁ Very Low certainty of evidence.

## Data Availability

No new data were generated or analyzed in support of this research.

## Supplementary Materials

The following supporting information can be downloaded at: https://www.mdpi.com/article/10.3390/cancers18071149/s1, Supplement S1: Search strategy: Ovid MEDLINE and Epub Ahead of Print, In-Process & Other Non-Indexed Citations and Daily <1946 to 2 August 2024>; Supplement S2: Search strategy and results: Embase <1988 to August 2024>; Supplement S3: Risk of bias summary and graph for overall survival (OS); Supplement S4: Risk of bias summary and graph for cognitive decline (CD); Supplement S5: PRISMA 2020 checklist.
