# Supplementary material for: The Trouble with Trials: Systematic Review and Meta-Analysis of Randomized Controlled Trials Comparing Stereotactic Radiosurgery, Whole Brain Radiotherapy, and Observation for Resected Metastatic Brain Disease
Source: Cancers (Basel). 2026 Apr 2;18(7):1149. doi: 10.3390/cancers18071149 (PMC13072340; doi:10.3390/cancers18071149)

Supplement S1. Search strategy: Ovid MEDLINE and Epub Ahead of Print, In-Process & Other Non-Indexed Citations and Daily <1946 to 2 August 2024>.

#	Searches	Results
1	exp brain neoplasms/sc	16181
2	exp brain neoplasms/	176923
3	((brain or cerebral or intracranial) adj5 metasta*).mp. [mp=title, abstract, original title, name of substance word, subject heading word, floating sub-heading word, keyword heading word, protocol supplementary concept word, rare disease supplementary concept word, unique identifier, synonyms]	27229
4	exp Neoplasm Metastasis/	225305
5	2 and 3	15956
6	1 or 3 or 5	32369
7	wbrt.mp.	2275
8	(srs.mp. or 7) and 6 [mp=title, abstract, original title, name of substance word, subject heading word, floating sub-heading word, keyword heading word, protocol supplementary concept word, rare disease supplementary concept word, unique identifier, synonyms]	3069
9	(stereotac* or "gamma knife" or radiosurg*).mp. [mp=title, abstract, original title, name of substance word, subject heading word, floating sub-heading word, keyword heading word, protocol supplementary concept word, rare disease supplementary concept word, unique identifier, synonyms]	46266
10	6 and 9	5709
11	whole brain radiotherapy.mp.	2489
12	6 and 11	1906
13	8 or 10	6376
14	13 and (random*.mp. or randomized controlled trial.pt.) [mp=title, abstract, original title, name of substance word, subject heading word, floating sub-heading word, keyword heading word, protocol supplementary concept word, rare disease supplementary concept word, unique identifier, synonyms]	637
15	13 and (blind* or mask*).mp. [mp=title, abstract, original title, name of substance word, subject heading word, floating sub-heading word, keyword heading word, protocol supplementary concept word, rare disease supplementary concept word, unique identifier, synonyms]	100
16	14 or 15	716

Supplement S2. Search strategy and results: Embase <1988 to August 2024>.

#	Searches	Results
1	brain metastasis/	46997
2	whole brain radiotherapy.mp. or whole brain radiotherapy/	5829
3	(wbrt or "whole brain radiation" or "whole brain irradiat*").mp. [mp=title, abstract, heading word, drug trade name, original title, device manufacturer, drug manufacturer, device trade name, keyword, floating subheading word, candidate term word]	7610
4	stereotactic radiosurgery.mp. or stereotactic radiosurgery/	15093
5	2 or 3	9387
6	4 or srs.mp. [mp=title, abstract, heading word, drug trade name, original title, device manufacturer, drug manufacturer, device trade name, keyword, floating subheading word, candidate term word]	29136
7	1 and (5 or 6)	8230
8	7 and (meta-analysis/ or systematic review/)	276
9	clinical trial/ or exp controlled clinical trial/	1885557
10	7 and 9	1167
11	8 or 10	815
12	remove duplicates from 11	1301
13	12 not case report/	768

Supplement S3. Risk of bias summary and graph for overall survival (OS).

Study	Random sequence generation	Allocation concealment	Blinding of participants and personnel	Blinding of outcome assessment	Incomplete outcome data	Selective reporting	Other	Overall risk
Patchell et al., 1998	+	^	*	+	*	+	+	High
Roos et al., 2006	+	^	*	+	*	+	+	High
Kocher et al., 2011	+	^	*	+	+	+	+	Moderate
Kayama et al., 2018	+	^	*	+	+	+	+	Moderate
Kepka et al., 2018	+	^	*	+	*	+	+	High
Brown et al., 2017	+	^	*	+	+	+	+	Moderate
Mahajan et al., 2017	+	^	*	+	+	+	+	Moderate

+ - Low risk of bias
* - Unclear risk of bias
^ - High risk of bias

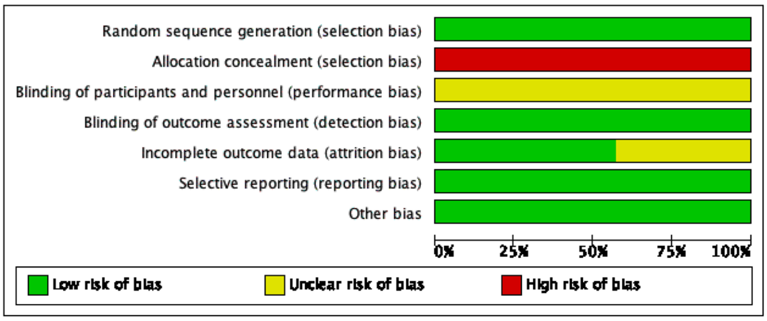

Supplement S4. Risk of bias summary and graph for cognitive decline (CD).

Study	Random sequence generation	Allocation concealment	Blinding of participants and personnel	Blinding of outcome assessment	Incomplete outcome data	Selective reporting	Other	Overall risk
Patchell et al., 1998	+	^	^	^	*	+	+	High
Roos et al., 2006	+	^	^	^	*	+	+	High
Kocher et al., 2011	+	^	^	^	+	+	+	High
Kayama et al., 2018	+	^	^	^	+	+	+	High
Kepka et al., 2018	+	^	^	^	*	+	+	High
Brown et al., 2017	+	^	+	+	+	+	+	Moderate

+ - Low risk of bias
* - Unclear risk of bias
^ - High risk of bias

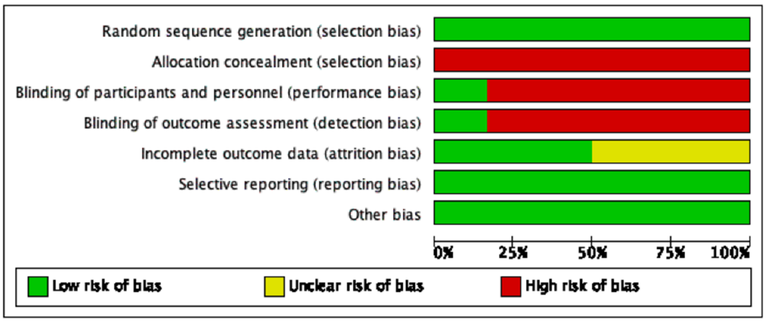

Supplement: Supplementary file 1 [file cancers-18-01149-s001.zip › cancers-4222689-supplementary/Supplements S1-S4.pdf]
